# Safety Assessment of Two New *Lactobacillus* Strains as Probiotic for Human Using a Rat Model

**DOI:** 10.1371/journal.pone.0159851

**Published:** 2016-07-28

**Authors:** Parisa Shokryazdan, Mohammad Faseleh Jahromi, Juan Boo Liang, Ramasamy Kalavathy, Chin Chin Sieo, Yin Wan Ho

**Affiliations:** 1 Institute of Tropical Agriculture, Universiti Putra Malaysia, 43400 UPM, Serdang, Selangor, Malaysia; 2 Faculty of Pharmacy, Universiti Teknologi MARA, 42300 Puncak, Alam, Selangor, Malaysia; 3 Institute of Bioscience, Universiti Putra Malaysia, 43400 UPM, Serdang, Selangor, Malaysia; Agricultural University of Athens, GREECE

## Abstract

Two previously isolated *Lactobacillus* strains (*L*. *fermentum* HM3 from human milk and *L*. *buchneri* FD2 from fermented dates), intended as probiotic for human, were assessed for their safety using acute and subacute oral toxicity tests in rats. In addition, their effects on cecal microflora and harmful bacterial enzymes (β-glucuronidase and β-glucosidase) of the tested animals were also determined. The results showed that *L*. *buchneri* FD2, *L*. *fermentum* HM3, or a mixture of them were safe up to a level of 10^10^ CFU/kg BW/day in a 14-day or 28-day treatment period. Both strains were well tolerated and there were no observed adverse effects on growth, feed consumption, cellular blood components and vital organs of the treated animals. The *Lactobacillus* strains were also able to reduce harmful intestinal bacterial enzymes, and decrease pathogenic bacterial populations while increasing beneficial bacterial populations. These results suggest that the two *Lactobacillus* strains are safe and could be potential probiotic for human.

## Introduction

The definition of probiotics, “live microorganisms that, when administered in adequate amounts, confer a health benefit on the host” [1], requires probiotic strains to be verified as “safe” because probiotic strains are administered alive and most of them are marketed as food supplements. Lactic acid bacteria, among them *Lactobacillus* strains, have been used extensively in food processing for a long time through human history. However, their scientific recognition is limited to the recent decades [2]. Although *Lactobacillus* strains are “Generally Recognized As Safe (GRAS)” [3], and the safety of these strains are seldom questioned, in recent years some species of this genus, and also from other genera of lactic acid bacteria, were isolated from some infective lesions [4–5] and these strains might be problematic. Since the safety aspect is very important for every probiotic strain, different methods, including *in vitro* studies, animal studies, and human clinical studies, have been used for assessing the safety of probiotic bacteria [6].

In the present study, two previously [7] isolated *Lactobacillus* strains (*L*. *fermentum* HM3 from human milk and *L*. *buchneri* FD2 from fermented dates) with the best probiotic attributes, based on the results of the *in vitro* studies, were selected as potential probiotics for human. The good probiotic traits of these two strains included high tolerance to acid and bile, strong ability to adhere to intestinal epithelial cells and good bioactivities. As these two are new isolated strains, and have no previous history of human consumption or food product use, they cannot automatically be regarded as “safe” sharing the same “GRAS” status as traditional *Lactobacillus* strains. It is necessary and essential to carry out safety assessment on these new strains as they are intended to be dietary or food supplements for human. Thus, in the present study, a safety assessment of the two *Lactobacillus* strains using acute and subacute oral toxicity tests in rats was conducted. In addition, their effects on cecal microflora and harmful bacterial enzymes (β-glucuronidase and β-glucosidase) of the tested animals were also determined.

## Materials and Methods

### *Lactobacillus* cultures (test products)

The two *Lactobacillus* strains, *L*. *fermentum* HM3 from human milk and *L*. *buchneri* FD2 from fermented date, were cultured separately in MRS broth medium (Merck, Germany) and incubated for 24 h at 37°C in anaerobic jars (Oxoid, UK) containing gaspack (AnaeroGen, Oxoid, UK). After incubation, the culture of each strain was centrifuged at 5000 × *g* for 10 min at 4°C. Supernatants were discarded and cell pellets were washed three times with deionized water. The cell pellets were freeze-dried and kept at -20°C until use. Cell suspensions used were freshly prepared from the freeze-dried stocks every day before gavaged to the animals. For cell suspension preparation, normal saline was used as diluent.

### Animals

For acute and subacute oral toxicity studies, 6- to 7-week-old male and female Sprague-Dawley rats were used. They were procured from A-Sapphire Enterprise, Selangor, Malaysia. The animals were separated according to gender and housed in wire-topped plastic cages (47 cm length × 35 cm width × 20 cm height) with appropriate space, and had free access to tap water and standard rodent diet (Specialty Feeds, Glen Forrest, WA, Australia). Both acute and subacute oral toxicity studies were approved by the Ethics Committee of the University Teknologi MARA, and in compliance with the National Research Council’s Guide for the Care and Use of Laboratory Animals [8].

### Acute oral toxicity study (single-dose toxicity study)

For the acute oral toxicity study, a total of 20 male and 20 female rats were used. The animals (of each gender) were randomly assigned to four groups of five rats each. After 7 days of acclimatization and fasting for 16 h, each group was administered (by oral gavage) a single dose of one of four treatments. The treatments were: i) 1 ml of normal saline (control), ii) 6 × 10^10^ CFU of *L*. *buchneri* FD2/kg body weight (BW) in 1 ml of normal saline (FD2), iii) 6 × 10^10^ CFU of *L*. *fermentum* HM3/kg BW in 1 ml of normal saline (HM3), and iv) 6 × 10^10^ CFU of a mixture of both *Lactobacillus* strains in 1 ml normal saline (1:1, v:v) (FD2+HM3). The rats were given standard rodent diet 3 h after oral gavage of treatments. They were then observed for signs of acute toxicity, changes on skin, fur, eyes and mucous membranes, somatomotor activity, behavior pattern, tremors, convulsions, salivation, diarrhea, lethargy, sleep, changes in gait, and mortality. Observations were made continuously for the first 4 h after treatment, then once daily for 14 days. All animals were weighed on the day of arrival, day 1 of treatment, day 7 and day 14. Feed residual was weighed daily, and the average feed consumption per animal was calculated. On day 15, the animals were anesthetized with diethyl-ether and macroscopic examination of the animals was carried out. This study was performed according to the OECD Guideline for the Testing of Chemicals No. 423, Acute Oral Toxicity, Acute Toxic Class Method, adopted December 17, 2001 [9].

### Subacute oral toxicity study (repeated-dose toxicity study)

#### Experimental design

The subacute oral toxicity study was a 28-day repeated-dose oral toxicity study. It was performed according to the OECD Guideline for the Testing of Chemicals No. 407, Repeated Dose 28-Day Oral Toxicity Study in Rodents, adopted on 3 October 2008 [10].

A total of 42 male and 42 female rats were used. The animals of each gender were randomly assigned to seven groups of six rats each. After acclimatization for 7 days, and fasting for 16 h, each group of animals received one of the following seven treatments: i) 1 ml of normal saline (control), ii) 1 × 10^9^ CFU of *L*. *buchneri* FD2/kg BW in 1 ml of normal saline [(FD2)L], iii) 1 × 10^10^ CFU of *L*. *buchneri* FD2/kg BW in 1 ml of normal saline [(FD2)H], iv) 1 × 10^9^ CFU of *L*. *fermentum* HM3/kg BW in 1 ml of normal saline [(HM3)L], v) 1 × 10^10^ CFU of *L*. *fermentum* HM3/kg BW in 1 ml of normal saline [(HM3)H], vi) 1 × 10^9^ CFU of a mixture of *L*. *buchneri* FD2 and *L*. *fermentum* HM3/kg BW in 1 ml of normal saline (1:1, v:v) [(FD2+HM3)L], vii) 1 × 10^10^ CFU of a mixture of *L*. *buchneri* FD2 and *L*. *fermentum* HM3/kg BW in 1 ml of normal saline (1:1, v:v) [(FD2+HM3)H]. Each treatment was administered (by oral gavage) once daily at 09:00 h for 28 consecutive days. The dose level of *Lactobacillus* strains at 1 × 10^9^ CFU/kg BW was designated as low dose (L) and that at 1 × 10^10^ CFU/kg BW was designated as high dose (H).

#### General observations

All general observations mentioned in the acute oral toxicity test were also followed for the subacute oral toxicity test, which included any changes in skin and fur, eyes and mucous membranes, respiratory, somatomotor activity, behavior pattern, tremors, convulsions, salivation, diarrhea, lethargy, sleep and changes in gait and posture. Physical parameters such as body weight, feed intake and local injuries, and mortality (if any), were recorded throughout the experimental period.

#### Hematological and serum biochemistry analyses

At the end of the experiment, animals were anesthetized with diethyl-ether, killed by cardiac puncture and blood was collected for hematology and serum biochemistry analyses. For hematological study, blood was collected in tubes containing K2EDTA and analyzed using an automated hematological analyzer (CELL-DYN 3700 Abbott Diagnostics, USA) for the following parameters: red blood cell count, hemoglobin, packed cell volume, mean corpuscular volume, mean corpuscular hemoglobin concentration, thrombocytes and white blood cell count (including lymphocytes, monocytes, neutrophils and eosinophils).

For serum biochemistry analysis, blood was collected in non-heparinized blood collection tubes, allowed to clot at room temperature, and centrifuged at 3000 × *g* for 10 min at 4°C after which serum was collected. Serum biochemistry was analyzed using an automatic clinical chemistry analyzer (Hitachi, Japan) for the following parameters: albumin, aspartate aminotransferase, alanine aminotransferase, alkaline phosphatase, total cholesterol, triglycerides, HDL-cholesterol, LDL-cholesterol, glucose, creatinine, total bilirubin, calcium, inorganic phosphorus, urea, total protein and electrolytes, including sodium, potassium and chloride.

#### Relative organ weights and histopathological examination

After sacrificing the animals, brain, heart, lung, liver, spleen, kidney, adrenal, thymus and intestine (jejunum) of male and female rats; testes, epididymis of male rats; and uterus and ovary of female rats were removed, and gross pathological examination was performed on all organs, including weights of organs, macroscopic appearance and presence of lesions. Relative weight of each organ was calculated as follows:

$$\text{Relative weight of organ }\left(\text{\%}\right)=\frac{\text{Weight of organ}}{\text{Live body weight}}\times 100$$

Histopathological examination was also performed on the brain, heart, lung, liver, spleen, kidney and jejunum of three male rats and three female rats from the high dose groups (1 × 10^10^ CFU of *Lactobacillus* strains) and control groups. Tissues of organs were fixed in 10% formalin, dehydrated for 16 h in an automatic tissue processor (Leica ASP 3000, Japan) and embedded in paraffin wax using a paraffin embedding system (Leica EG 1160, Japan). Each sample was cut to 4 μm-thick sections using a rotary microtome (Leica RM 2155, Japan). The sections were fixed on a glass slide, heated at 57°C until dried, stained with haematoxylin and eosin [11] and examined under a light microscope (Dialux, Leitz Wetzlar, Germany).

#### Microbiological analysis of cecal contents

Freshly collected cecal contents of rats were used immediately for enumeration of lactobacilli, bifidobacteria, *E*. *coli*, *Salmonella* and total aerobes using the conventional microbiological (spread plate) method. Briefly, 1 g of cecal content was suspended in 9 ml PBS and vortexed for 1 min. Samples were serially diluted in sterile diluents (0.5% peptone water in distilled water) and 100 μl of 10^−4^ to 10^−6^ dilutions were streaked on appropriate selective media for enumeration of different groups of bacteria. MRS agar was used for enumeration of lactobacilli, Bifidus Selective agar for bifidobacteria, Brain-Heart Infusion agar for total aerobes, Brilliant Green agar for *Salmonella* and Eosin Methylene Blue agar for *E*. *coli* (all media from Sigma, USA, except MRS from Merck, Germany). After incubation in appropriate conditions for each group of bacteria (72 h at 37°C in anaerobic condition for lactobacilli and bifidobacteria, and 48 h at 39°C in aerobic condition for *Salmonella*, *E*. *coli* and total aerobes), colonies on the plates were counted and microbial population was expressed as log CFU/g cecal content.

#### Harmful intestinal bacterial enzyme assay

Cecal contents freshly collected from the rats were also used immediately for analysis of harmful intestinal bacterial enzymes (β-glucosidase and β-glucuronidase). One gram of cecal content was suspended in 10 ml of PBS (pH 7.2), then centrifuged at 3000 × *g* for 5 min at room temperature. Supernatants were used for enzyme assays. The assays for β-glucosidase and β-glucuronidase activities were carried out according to the method described by Lee *et al*. [12] with modifications. Briefly, 0.8 ml of 2 mM p-nitrophenyl-β-D-glucopyranoside (Sigma) (for β-glucosidase activity) or 2 mM p-nitrophenyl-β-D-glucuronide (Sigma) (for β-glucuronidase activity) and 0.2 ml of sample were incubated at 37°C for 1 h. The reaction was stopped by adding 1 ml of 0.5 mol l^-1^ NaOH, and the mixture was centrifuged at 4000 × g for 10 min at room temperature. The β-glucosidase and β-glucuronidase activities of the supernatant was determined by measuring absorbance at 405 nm using a spectrophotometer. Different concentrations (0, 0.1, 0.2, 0.5, 1 and 10 mmol l^-1^) of p-nitrophenol (Sigma) were used for preparation of a standard curve. The enzyme activity was expressed as unit g^-1^ cecal contents. One unit is defined as the activity required to release 1 μmol l^-1^ of p-nitrophenol in 1 h.

### Statistical analysis

Experimental data were analyzed by one-way ANOVA procedure of SAS program (2008) version 9.2. followed by multiple comparison among the means using Duncan’s new multiple range test. Differences were considered significant if P < 0.05.

## Results

### Single-dose acute oral toxicity study

The results of the 14-day single-dose acute oral toxicity study in rats showed that a single oral dose of 6 × 10^10^ CFU *L*. *buchneri* FD2, *L*. *fermentum* HM3 or a mixture of both strains/kg BW did not cause mortality or treatment-related toxicity signs in any of the animals. There was no change in appearance or behavior and there was also no weight loss or loss in feed intake (no significant difference between control and treatment rats) (Table 1). At necropsy, all organs examined in both male and female rats were free from any gross pathological changes. Thus, based on the OECD/OCDE guideline No. 423, there was no need to carry out histopathological examination. Generally, there was no evidence of any toxicity in rats given *L*. *buchneri* FD2, *L*. *fermentum* HM3 or a mixture of the two *Lactobacillus* strains in the acute oral toxicity study.

**Table 1:** Weekly body weight gain, daily feed intake and mortality of male and female rats in the acute oral toxicity study.

	Male rats^1^				Female rats^1^			
	Control	FD2	HM3	FD2+HM3	Control	FD2	HM3	FD2+HM3
**Weekly weight gain (g)**	38.83±7.08	44.17±3.51	39.50±1.58	42.10±4.66	12.38±2.02	12.80±2.08	12.38±1.89	12.88±1.55
**Daily feed intake (g)**	19.49±2.24	19.20±1.96	19.34±2.39	19.58±2.25	13.47±2.08	13.45±1.88	13.77±2.46	13.10±1.80
**Mortality (%)**	0.00	0.00	0.00	0.00	0.00	0.00	0.00	0.00
**Toxicity signs**	None	None	None	None	None	None	None	None

^1^Values are mean ± SD of 6 replicates

Control, normal saline; FD2, normal saline + 6 × 10^10^ CFU of *L*. *buchneri* FD2/kg BW; HM3, normal saline + 6 × 10^10^ CFU of *L*. *fermentum* HM3/kg BW; FD2+HM3, normal saline + 6 × 10^10^ CFU of a mixture (1:1, w:w) of *L*. *buchneri* FD2 and *L*. *fermentum* HM3

### Repeated-dose subacute oral toxicity study

#### General signs

*Lactobacillus buchneri* FD2, *L*. *fermentum* HM3 and their mixture (1:1, w:w) were administered by oral gavage at doses of 1×10^10^ CFU/kg BW/day (high dose) and 1×10^9^ CFU/kg BW/day (low dose) for 28 consecutive days. No death or treatment-related sign of toxicity was observed in any of the animals throughout the 28-day experimental period. Appearance and behavior of the animals were similar for all groups throughout the study.

#### Body weight and feed consumption

The results of body weights of male and female rats are shown in Tables 2 and 3, respectively. Body weights of control and treatment male rats were not significantly different from days 1 to 14 of the experiment. However, at day 21, the body weights of male rats receiving high and low doses of FD2, high dose of HM3 and low dose of FD2+HM3 were significantly (P < 0.05) higher than that of control, and at day 28, the body weights of all male rats receiving the *Lactobacillus* strains treatments were significantly (P < 0.05) more than the control.

**Table 2:** Body weights of male rats.

Treatment	Body weight (g) ^1^				
	Day 1	Day 7	Day 14	Day 21	Day 28
**Control**	219.7±13.4	246.0±10.2	265.8±9.9	279.8±13.0 ^b^	288.8±15.9 ^b^
**(FD2)H**	231.2±8.6	267.0±15.0	288.2±14.4	303.0±14.8 ^a^	312.2±13.3 ^a^
**(FD2)L**	228.2±11.5	263.3±13.3	288.7±14.2	304.7±5.8 ^a^	316.7±18.4 ^a^
**(HM3)H**	227.7±20.9	259.3±12.2	282.3±8.8	298.5±9.8 ^a^	312.0±12.0 ^a^
**(HM3)L**	211.8±19.9	248.0±18.5	274.3±15.0	295.3±12.7 ^a^^b^	311.3±12.0 ^a^
**(HM3+FD2)H**	214.7±18.6	250.5±20.8	272.0±20.3	289.4±17.3 ^a^^b^	305.2±14.3 ^a^
**(HM3+FD2)L**	235.3±2.7	261.0±4.9	281.7±9.0	297.9±9.6 ^a^	307.7±11.3 ^a^

^1^Values are mean ± SD of 6 replicates

^a–b^ Means within a column with no common superscript differ significantly (P < 0.05)

Control, normal saline; (FD2)L, normal saline + 1 × 10^9^ CFU of *L*. *buchneri* FD2/kg BW; (FD2)H, normal saline + 1 × 10^10^ CFU of *L*. *buchneri* FD2/kg BW; (HM3)L, normal saline + 1 × 10^9^ CFU of *L*. *fermentum* HM3/kg BW; (HM3)H, normal saline + 1 × 10^10^ CFU of *L*. *fermentum* HM3/kg BW; (FD2+HM3)L, normal saline + 1 × 10^9^ CFU of a mixture (1:1, w:w) of *L*. *buchneri* FD2 and *L*. *fermentum* HM3; (FD2+HM3)H, normal saline + 1 × 10^10^ CFU of a mixture (1:1, w:w) of *L*. *buchneri* FD2 and *L*. *fermentum* HM3

**Table 3:** Body weights of female rats.

Treatment	Body weight (g) ^1^				
	Day 1	Day 7	Day 14	Day 21	Day 28
**Control**	185.3±10.8	198.0±9.7	208.3±8.8	214.5±7.8	219.7±7.6
**(FD2)H**	179.3±11.4	191.8±13.4	200.2±13.4	206.0±14.0	210.7±14.7
**(FD2)L**	183.0±10.3	194.8±12.7	202.0±13.9	207.3±15.3	211.7±14.8
**(HM3)H**	179.2±19.4	191.2±18.8	198.3±18.5	204.5±18.2	210.3±18.3
**(HM3)L**	179.8±18.3	193.3±18.2	202.8±19.3	210.8±21.3	217.5±22.0
**(HM3+FD2)H**	178.2±14.6	192.7±16.7	201.0±18.1	209.5±19.0	214.8±19.4
**(HM3+FD2)L**	172.2±6.5	184.8±8.5	196.3±12.5	205.5±15.8	210.7±17.4

^1^Values are mean ± SD of 6 replicates

Control, normal saline; (FD2)L, normal saline + 1 × 10^9^ CFU of *L*. *buchneri* FD2/kg BW; (FD2)H, normal saline + 1 × 10^10^ CFU of *L*. *buchneri* FD2/kg BW; (HM3)L, normal saline + 1 × 10^9^ CFU of *L*. *fermentum* HM3/kg BW; (HM3)H, normal saline + 1 × 10^10^ CFU of *L*. *fermentum* HM3/kg BW; (FD2+HM3)L, normal saline + 1 × 10^9^ CFU of a mixture (1:1, w:w) of *L*. *buchneri* FD2 and *L*. *fermentum* HM3; (FD2+HM3)H, normal saline + 1 × 10^10^ CFU of a mixture (1:1, w:w) of *L*. *buchneri* FD2 and *L*. *fermentum* HM3

Mean body weights of female rats were not significantly different between the control and those given high or low doses of FD2, HM3 or FD2+HM3 throughout the experimental period (Table 3). The results of daily feed consumption of male and female rats are shown in Table 4. In both male and female rats there were no significant differences between control rats and those given high or low doses of FD2, HM3 or FD2+HM3 throughout the experimental period.

**Table 4:** Feed consumption of male and female rats.

	Feed consumption of male rats (g/rat/day)^1^				Feed consumption of female rats (g/rat/day)^1^			
Treatment	Day 1 to 7	Day 8 to 14	Day 15 to 21	Day 22 to 28	Day1 to 7	Day 8 to 14	Day 15 to 21	Day 22 to 28
**Control**	27.0±1.6	25.9±0.9	24.4±2.0	24.8±0.5	17.1±0.7	17.5±1.1	17.2±1.2	16.9±0.7
**(FD2)H**	25.2±1.4	24.9±1.8	24.1±1.7	24.9±0.8	17.7±1.3	17.8±1.2	17.1±1.8	18.0±0.9
**(FD2)L**	27.4±1.7	26.1±1.4	24.7±2.2	26.0±1.0	17.4±1.2	17.5±0.7	16.3±1.7	16.2±0.7
**(HM3)H**	27.5±1.4	25.6±2.0	24.4±1.6	25.1±0.5	18.3±1.0	18.3±0.6	17.6±2.0	17.4±0.6
**(HM3)L**	25.8±1.1	26.5±1.6	25.0±1.8	25.2±1.0	17.3±1.5	16.6±1.1	16.6±1.7	16.3±0.4
**(HM3+FD2)H**	26.1±1.1	25.0±1.0	24.7±1.4	25.9±1.1	18.4±1.1	18.6±1.3	17.5±1.8	18.6±0.8
**(HM3+FD2)L**	26.5±1.7	26.7±1.2	25.8±1.9	26.2±0.4	18.4±1.1	18.0±0.8	17.2±1.7	17.3±0.7

^1^Values are mean ± SD of 6 replicates

Control, normal saline; (FD2)L, normal saline + 1 × 10^9^ CFU of *L*. *buchneri* FD2/kg BW; (FD2)H, normal saline + 1 × 10^10^ CFU of *L*. *buchneri* FD2/kg BW; (HM3)L, normal saline + 1 × 10^9^ CFU of *L*. *fermentum* HM3/kg BW; (HM3)H, normal saline + 1 × 10^10^ CFU of *L*. *fermentum* HM3/kg BW; (FD2+HM3)L, normal saline + 1 × 10^9^ CFU of a mixture (1:1, w:w) of *L*. *buchneri* FD2 and *L*. *fermentum* HM3; (FD2+HM3)H, normal saline + 1 × 10^10^ CFU of a mixture (1:1, w:w) of *L*. *buchneri* FD2 and *L*. *fermentum* HM3

#### Relative organ weights and histopathological examination

No gross pathological changes were found in rats of all the groups. The relative organ weights of brain, heart, lung, liver, spleen, kidney, adrenal, thymus for male and female rats; testes and epididymis for male rats; and uterus and ovary for female rats were not significantly different (P > 0.05) between the control and all treated groups (Tables 5 and 6 for male and female rats, respectively).

**Table 5:** Relative organ weights (%) of male rats^1^.

Treatment	Heart	Lung	Liver	Kidney	Spleen	Intestine	Brain	Adrenal	Thymus	Testis	Epididymis
**Control**	0.349±0.051	0.857±0.172	3.059±0.371	0.377±0.056	0.184±0.018	6.192±0.509	0.587±0.056	0.020±0.002	0.148±0.034	0.473±0.046	0.355±0.053
**(FD2)H**	0.289±0.038	0.776±0.1	2.682±0.408	0.336±0.053	0.163±0.029	5.694±0.611	0.574±0.045	0.017±0.002	0.135±0.014	0.477±0.051	0.336±0.0320
**(FD2)L**	0.327±0.041	0.795±0.080	2.878±0.274	0.339±0.036	0.179±0.029	6.302±0.200	0.563±0.023	0.017±0.002	0.123±0.018	0.479±0.052	0.335±0.048
**(HM3)H**	0.319±0.020	0.725±0.094	2.944±0.227	0.324±0.041	0.191±0.034	6.242±0.829	0.557±0.025	0.017±0.003	0.116±0.032	0.457±0.054	0.352±0.061
**(HM3)L**	0.332±0.050	0.814±0.033	2.959±0.471	0.364±0.054	0.192±0.023	6.323±1.165	0.591±0.039	0.018±0.004	0.128±0.019	0.523±0.075	0.357±0.031
**(HM3+FD2)H**	0.305±0.024	0.711±0.153	2.964±0.176	0.348±0.024	0.176±0.025	6.056±0.554	0.578±0.024	0.017±0.002	0.141±0.010	0.479±0.039	0.316±0.0250
**(HM3+FD2)L**	0.304±0.021	0.730±0.133	2.923±0.292	0.333±0.036	0.167±0.020	5.598±0.921	0.559±0.043	0.018±0.004	0.139±0.022	0.507±0.075	0.376±0.066

^1^Values are mean ± SD of 6 replicates

Control, normal saline; (FD2)L, normal saline + 1 × 10^9^ CFU of *L*. *buchneri* FD2/kg BW; (FD2)H, normal saline + 1 × 10^10^ CFU of *L*. *buchneri* FD2/kg BW; (HM3)L, normal saline + 1 × 10^9^ CFU of *L*. *fermentum* HM3/kg BW; (HM3)H, normal saline + 1 × 10^10^ CFU of *L*. *fermentum* HM3/kg BW; (FD2+HM3)L, normal saline + 1 × 10^9^ CFU of a mixture (1:1, w:w) of *L*. *buchneri* FD2 and *L*. *fermentum* HM3; (FD2+HM3)H, normal saline + 1 × 10^10^ CFU of a mixture (1:1, w:w) of *L*. *buchneri* FD2 and *L*. *fermentum* HM3

**Table 6:** Relative organ weights (%) of female rats^1^.

Treatment	Heart	Lung	Liver	Kidney	Spleen	Intestine	Brain	Adrenal	Thymus	Uterus	Ovary
**Control**	0.301±0.022	0.673±0.050	3.162±0.549	0.280±0.034	0.215±0.022	5.489±1.064	0.732±0.058	0.031±0.005	0.172±0.017	0.197±0.047	0.053±0.013
**(FD2)H**	0.321±0.044	0.673±0.120	3.098±0.536	0.317±0.054	0.194±0.041	6.565±0.830	0.739±0.135	0.031±0.006	0.173±0.017	0.257±0.024	0.054±0.005
**(FD2)L**	0.287±0.049	0.577±0.117	2.934±0.577	0.284±0.050	0.190±0.027	5.962±0.936	0.738±0.103	0.033±0.005	0.188±0.044	0.225±0.033	0.055±0.012
**(HM3)H**	0.326±0.048	0.680±0.065	2.984±0.404	0.304±0.043	0.192±0.021	6.370±1.114	0.788±0.116	0.031±0.005	0.165±0.036	0.259±0.034	0.055±0.011
**(HM3)L**	0.290±0.029	0.660±0.132	3.057±0.531	0.324±0.072	0.179±0.014	6.334±0.941	0.765±0.107	0.030±0.007	0.174±0.038	0.238±0.048	0.048±0.008
**(HM3+FD2)H**	0.316±0.026	0.621±0.090	3.092±0.410	0.311±0.038	0.190±0.024	5.636±0.902	0.790±0.071	0.030±0.004	0.164±0.044	0.258±0.043	0.051±0.006
**(HM3+FD2)L**	0.307±0.051	0.632±0.060	3.102±0.599	0.304±0.035	0.186±0.037	6.649±1.180	0.810±0.147	0.033±0.007	0.172±0.023	0.261±0.033	0.052±0.003

^1^Values are mean ± SD of 6 replicates

Control, normal saline; (FD2)L, normal saline + 1 × 10^9^ CFU of *L*. *buchneri* FD2/kg BW; (FD2)H, normal saline + 1 × 10^10^ CFU of *L*. *buchneri* FD2/kg BW; (HM3)L, normal saline + 1 × 10^9^ CFU of *L*. *fermentum* HM3/kg BW; (HM3)H, normal saline + 1 × 10^10^ CFU of *L*. *fermentum* HM3/kg BW; (FD2+HM3)L, normal saline + 1 × 10^9^ CFU of a mixture (1:1, w:w) of *L*. *buchneri* FD2 and *L*. *fermentum* HM3; (FD2+HM3)H, normal saline + 1 × 10^10^ CFU of a mixture (1:1, w:w) of *L*. *buchneri* FD2 and *L*. *fermentum* HM3

#### Histopathological examination

**Fig 1:**
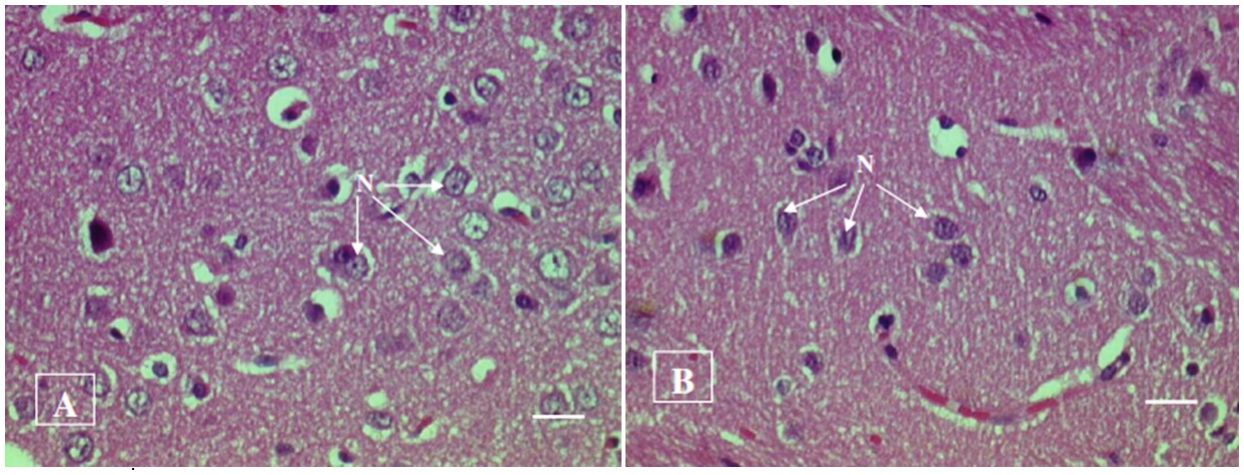
Light micrograph of the brain (cerebrum) of (A) a control rat, and (B) a rat given a mixture of *L*. *buchneri* FD2 and *L*. *fermentum* HM3, showing normal nerve cells (N) with a distinct nucleus. No inflammatory or cellular changes were observed in the brain of the treated rat. Bar = 20 μm.

**Fig 2:**
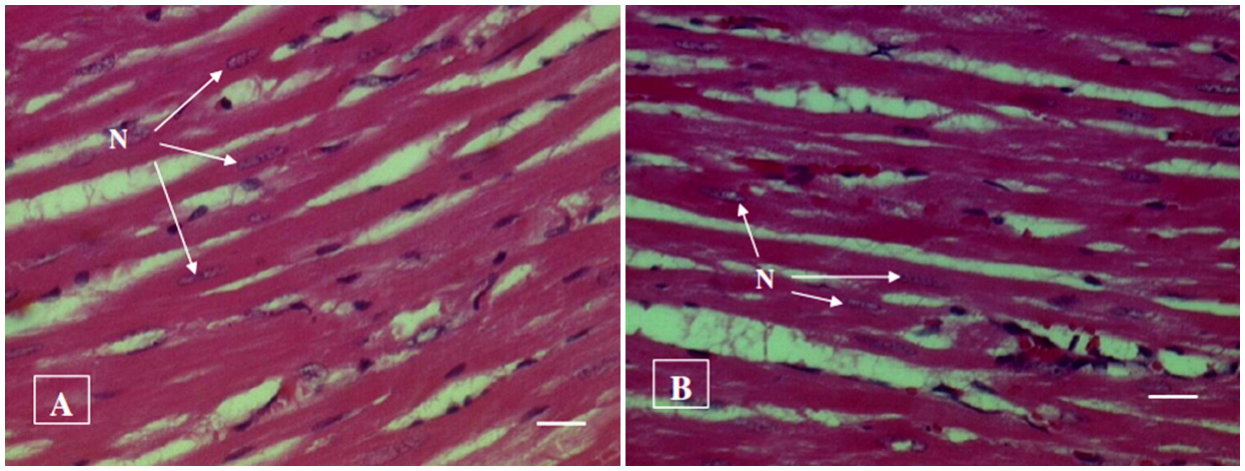
Light micrograph of cardiac muscle fibers of (A) a control rat, and (B) a rat given a mixture of *L*. *buchneri* FD2 and *L*. *fermentum* HM3, showing normal histological structure of cardiac muscle fibers with their nuclei (N). No inflammatory or cellular changes were observed in the heart of the treated rat. Bar = 20 μm.

**Fig 3:**
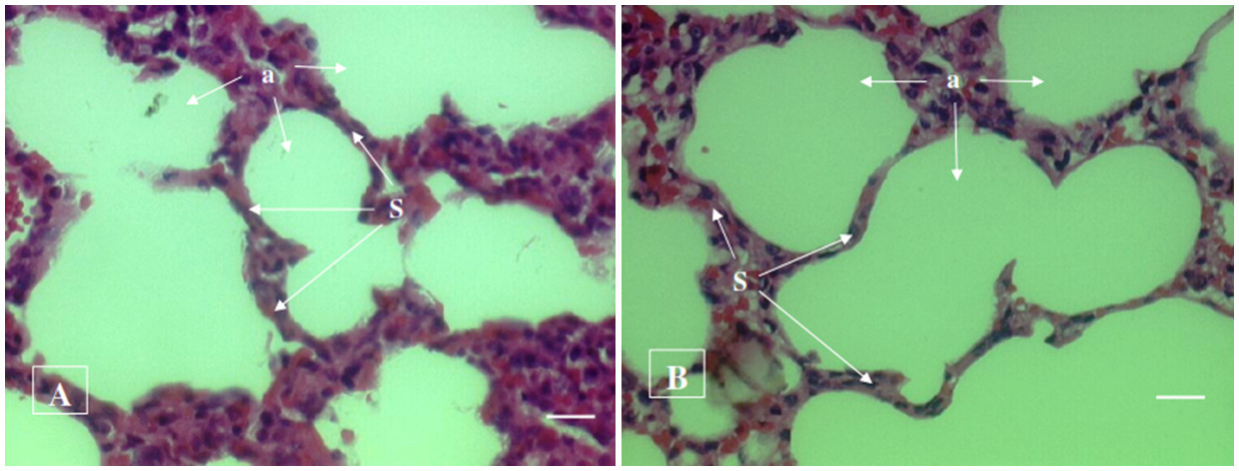
Light micrograph of the lung of (A) a control rat, and (B) a rat given a mixture of *L*. *buchneri* FD2 and *L*. *fermentum* HM3, showing normal histological structure of lung comprising of alveolus (a) and inter-alveolar septum (S). No inflammatory or cellular changes were observed in the lung of the treated rat. Bar = 20 μm.

**Fig 4:**
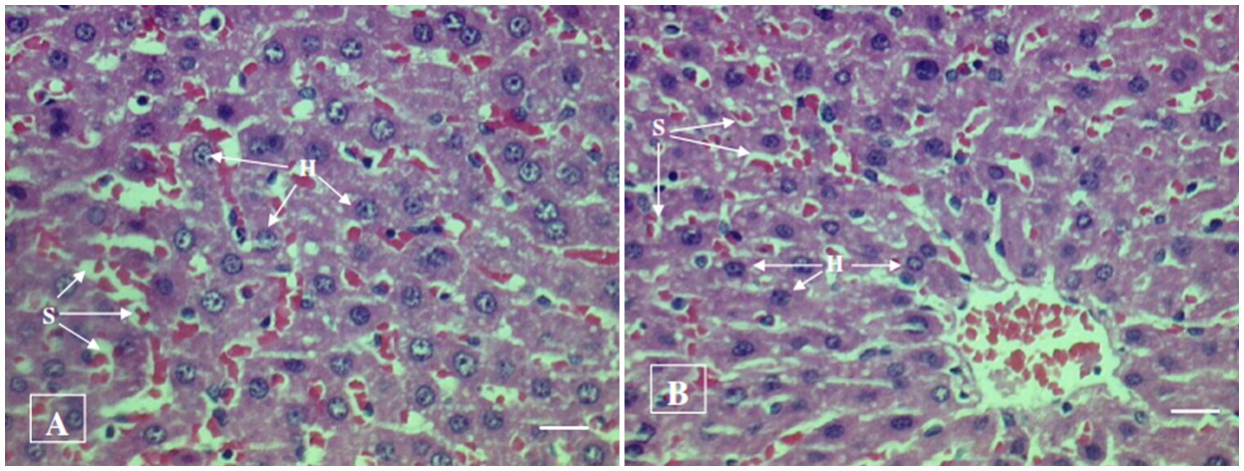
Light micrograph of the liver of (A) a control rat, and (B) a rat given a mixture of *L*. *buchneri* FD2 and *L*. *fermentum* HM3, showing normal histological structure of hepatocytes (H) and sinusoids (S). No inflammatory or cellular changes were observed in the liver of the treated rat. Bar = 20 μm.

**Fig 5:**
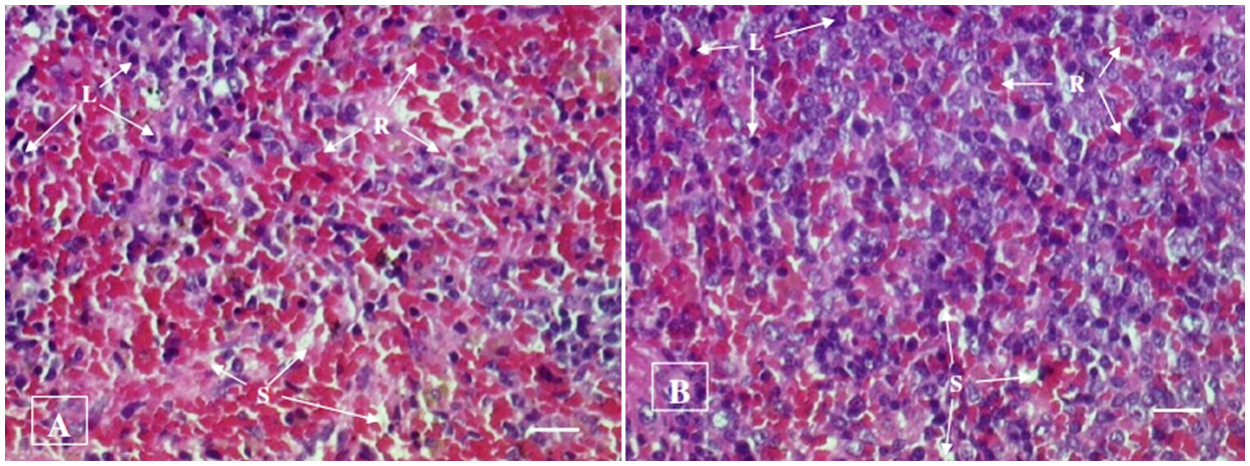
Light micrograph of the spleen of (A) a control rat, and (B) a rat given a mixture of *L*. *buchneri* FD2 and *L*. *fermentum* HM3, showing normal histological structure of spleen comprising of sinusoids (S), lymphocytes (L) and red blood cells (R). No inflammatory or cellular changes were observed in the spleen of the treated rat. Bar = 20 μm.

**Fig 6:**
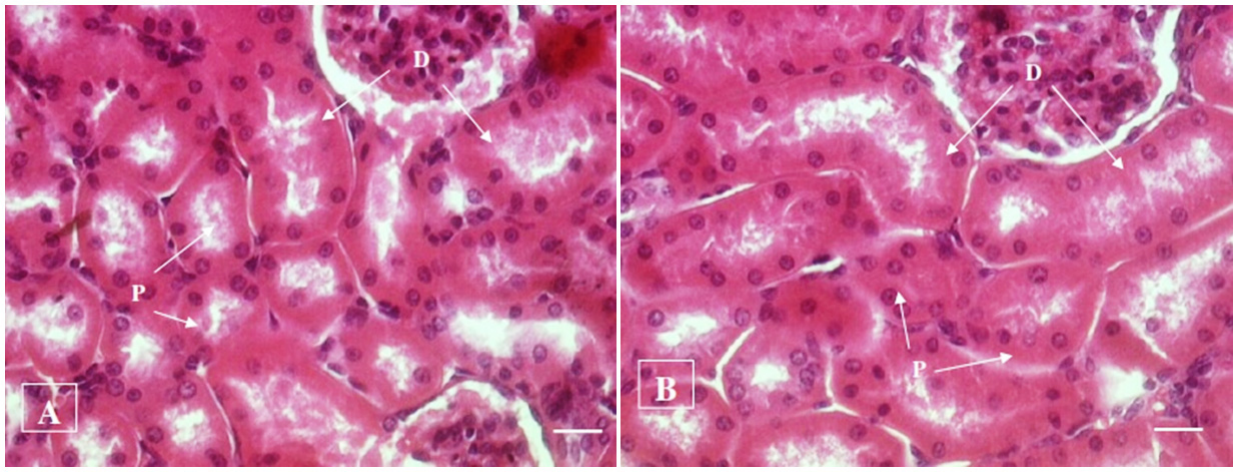
Light micrograph of the kidney of a (A) control rat, and (B) a rat given a mixture of *L*. *buchneri* FD2 and *L*. *fermentum* HM3, showing normal histological structure of kidney comprising of proximal (P) and distal (D) convoluted tubules. No inflammatory or cellular changes were observed in the kidney of the treated rat. Bar = 20 μm.

**Fig 7:**
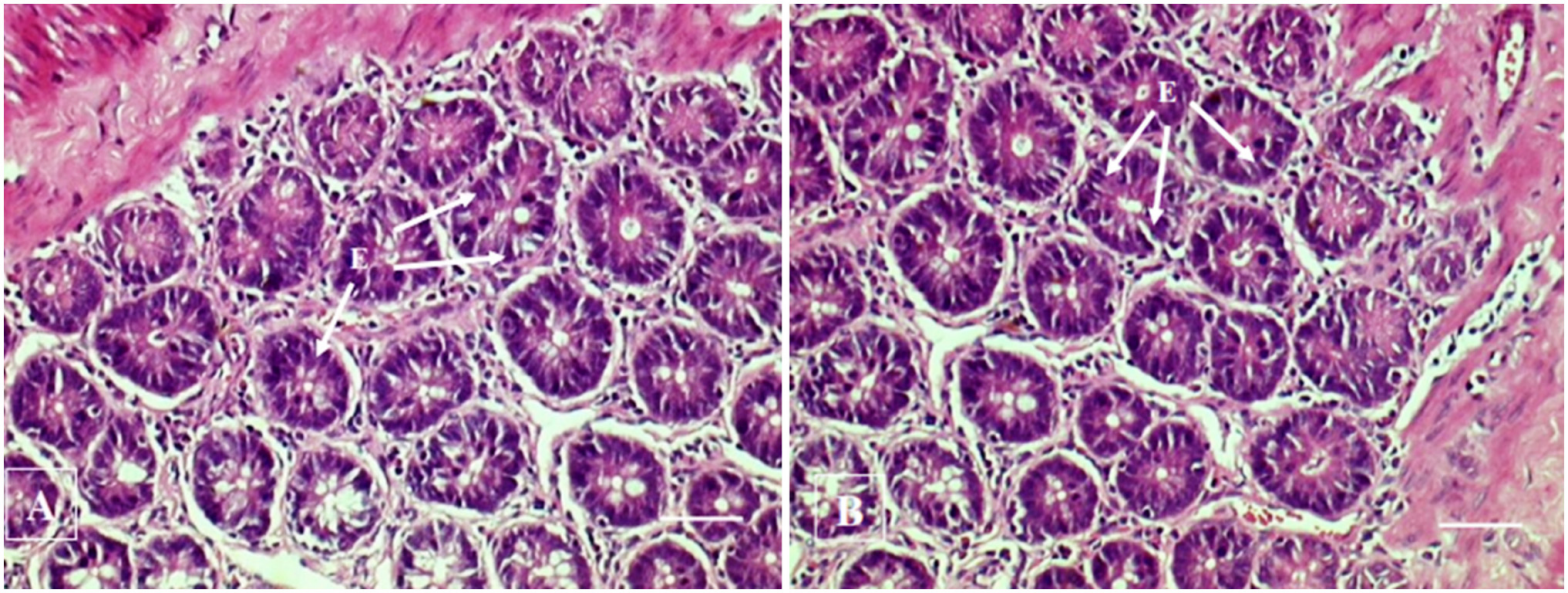
Light micrograph of the intestine of (A) a control rat, and (B) a rat given a mixture of *L*. *buchneri* FD2 and *L*. *fermentum* HM3, showing normal histological structure of the small intestine (jejunum) comprising of epithelial cells (E). No inflammatory or cellular changes were observed in the intestine of the treated rat. Bar = 50 μm.

No histopathological abnormalities or changes were observed in all the groups of animals. No necrosis, fibrosis, loss of normal architecture, atrophy or inflammation was observed in any of the examined organs i.e. brain, heart, lung, liver, spleen, kidney and small intestine in the control and treated groups of both male and female rats. Figs 1–7 show representatives of light micrographs of brain, heart, lung, liver, spleen, kidney and small intestine of rats in the control and treated groups.

#### Hematology and serum biochemistry analysis

Results of hematological parameters measured such as red blood cell count, hemoglobin, packed cell volume, mean corpuscular volume, mean corpuscular hemoglobin concentration, thrombocytes and white blood cell count (including lymphocytes, monocytes, neutrophils and eosinophils) showed that there were no significant differences between the control and treated groups in both male and female rats (Table 7).

**Table 7:** Hematological parameters of male and female rats^1^.

**Treatment (male rats)**	**RBC (×10**^**12**^**/L)**	**HB (g/L)**	**PCV (L/L)**	**MCV (fL)**	**MCHC (g/L)**	**WBC (×10**^**9**^**/L)**	**Neut. (%)**	**Lymp. (%)**	**Mono. (%)**	**Eosin. (%)**	**Thrombo. (×10**^**9**^**/L)**
**Control**	8.2±0.4	152.2±12.2	0.45±0.03	55.0±2.3	336.5±6.8	14.6±4.0	18.60±4.67	72.8±4.3	3.6±0.6	1.60±0.89	986.6±140.7
**(FD2)H**	8.1±0.2	149.8±5.8	0.45±0.01	55.4±1.8	333.0±12.1	12.8±2.5	19.00±4.18	71.2±5.4	5.2±1.8	0.60±0.55	810.8±183.5
**(FD2)L**	8.1±0.4	149.8±3.8	0.45±0.01	55.9±1.4	331.8±3.4	12.1±4.2	22.00±3.46	68.7±3.8	3.3±0.6	0.83±0.75	962.8±156.7
**(HM3)H**	8.0±0.8	148.0±8.3	0.44±0.02	55.3±4.0	336.4±5.7	16.7±5.4	21.00±2.76	67.8±5.8	4.4±2.1	2.17±1.94	962.5±343.0
**(HM3)L**	8.1±0.3	153.2±4.0	0.46±0.01	56.0±1.6	336.7±6.4	16.4±2.7	17.33±1.53	74.0±2.5	4.2±0.6	1.83±0.98	943.0±246.1
**(HM3+FD2)H**	7.9±0.2	149.2±6.8	0.44±0.01	56.2±1.6	335.9±7.5	10.9±3.2	22.40±5.94	67.6±5.9	4.6±0.8	1.40±0.55	959.4±94.0
**(HM3+FD2)L**	7.8±0.6	145.4±9.1	0.44±0.03	56.8±1.6	330.5±4.3	9.3±5.2	22.60±5.22	68.2±4.7	4.2±1.2	1.00±1.22	874.2±154.0
**Treatment (male rats)**	RBC (×10^12^/L)	HB (g/L)	PCV (L/L)	MCV (fL)	MCHC (g/L)	WBC (×10^9^/L)	Neut. (%)	Lymp. (%)	Mono. (%)	Eosin. (%)	Thrombo. (×10^9^/L)
**Control**	7.6±0.4	140.0±4.9	0.42±0.02	54.8±0.8	337.4±5.1	12.1±3.4	22.40±8.20	76.34±7.9	4.2±1.8	1.0±0.0	1243.3±142.8
**(FD2)H**	7.4±0.4	140.3±4.4	0.41±0.01	55.7±1.7	339.5±6.2	11.8±3.1	23.67±7.42	75.17±11.5	4.2±2.4	1.5±0.7	1187.5±151.0
**(FD2)L**	7.7±0.5	140.7±2.0	0.42±0.01	53.7±3.1	339.0±6.7	12.7±2.8	25.83±7.63	72.23±6.4	4.0±1.1	1.5±0.6	1373.2±100.2
**(HM3)H**	7.6±0.4	142.8±6.8	0.42±0.02	55.4±1.1	340.1±3.8	13.6±2.9	21.17±8.64	77.55±11.4	5.2±1.9	1.0±0.0	1256.8±91.8
**(HM3)L**	7.6±0.4	140.0±4.9	0.42±0.02	54.8±0.8	337.4±5.1	12.1±3.4	22.17±7.03	76.45±8.5	5.2±1.0	1.5±0.6	1243.3±142.8
**(HM3+FD2)H**	7.7±0.3	142.8±2.6	0.42±0.01	54.9±2.3	337.5±8.7	11.9±1.6	26.17±4.88	70.77±5.3	5.2±1.0	1.8±0.5	1218.2±224.9
**(HM3+FD2)L**	7.7±0.4	141.7±6.2	0.42±0.02	54.2±1.7	341.3±2.9	10.1±1.6	26.17±7.63	70.58±10.5	4.8±1.2	1.8±1.0	1259.2±91.7

^1^Values are mean ± SD of 6 replicates

Control, normal saline; (FD2)L, normal saline + 1 × 10^9^ CFU of *L*. *buchneri* FD2/kg BW; (FD2)H, normal saline + 1 × 10^10^ CFU of *L*. *buchneri* FD2/kg BW; (HM3)L, normal saline + 1 × 10^9^ CFU of *L*. *fermentum* HM3/kg BW; (HM3)H, normal saline + 1 × 10^10^ CFU of *L*. *fermentum* HM3/kg BW; (FD2+HM3)L, normal saline + 1 × 10^9^ CFU of a mixture (1:1, w:w) of *L*. *buchneri* FD2 and *L*. *fermentum* HM3; (FD2+HM3)H, normal saline + 1 × 10^10^ CFU of a mixture (1:1, w:w) of *L*. *buchneri* FD2 and *L*. *fermentum* HM3; fL, femtoliters or 10^-15^L; RBC, red blood cell count; HB, hemoglobin; PCV, packed cell volume; MCV, mean corpuscular volume; MCHC, mean corpuscular hemoglobin concentration; Thrombo., thrombocytes; WBC, white blood cell count; Neut., neutrophils; Lymp., lymphocytes; Mono., monocytes; Eosin., eosinophils.

Results of the serum biochemical parameters such as albumin, aspartate aminotransferase (AST), alanine aminotransferase (ALT), alkaline phosphatase (ALP), total cholesterol, triglycerides, HDL-cholesterol, LDL-cholesterol, glucose, creatinine, total bilirubin, calcium, inorganic phosphorus, urea, total protein and electrolytes, including sodium, potassium and chloride, also showed that there were no significant differences between the control and treated groups in male and female rats (Tables 8 and 9, respectively).

**Table 8:** Serum biochemical parameters of male rats^1^.

**Treatment**	**ALB (g/L)**	**ALT (U/L)**	**AST (U/L)**	**ALP (U/L)**	**Creat. (μmol/L)**	**Glu. (mmol/L)**	**T. bil. (μmol/L)**	**Urea (mmol/L)**	**T. prot. (g/L)**
**Control**	41.9±5.6	65.0±7.9	138.1±2.4	169.0±26.7	53.8±5.3	7.8±0.7	2.0±1.1	6.6±0.5	55.7±5.2
**(FD2)H**	36.3±2.3	60.6±6.8	134.9±9.4	159.5±44.7	57.7±4.1	6.8±1.4	0.9±0.7	6.7±0.9	56.0±7.1
**(FD2)L**	39.4±7.1	73.7±15.3	156.4±23.9	159.7±22.8	55.0±1.7	7.3±1.0	0.9±0.4	7.3±1.4	55.7±4.5
**(HM3)H**	39.3±4.1	63.5±11.6	140.4±23.1	150.0±23.3	57.5±3.3	7.5±1.1	1.3±0.4	7.4±0.9	60.7±4.5
**(HM3)L**	36.4±3.2	66.8±12.7	139.7±21.4	143.3±19.8	54.3±5.3	7.4±0.9	1.3±0.3	6.5±0.7	55.1±4.3
**(HM3+FD2)H**	42.8±4.5	78.5±18.0	157.1±14.0	155.2±42.8	55.5±3.6	6.3±0.5	0.9±0.7	6.5±0.4	59.1±5.8
**(HM3+FD2)L**	38.9±2.3	73.8±10.8	154.2±6.1	190.3±62.3	56.7±3.9	7.1±1.0	0.9±0.7	6.5±0.8	56.0±3.3
**Treatment**	**T. chol. (mmol/L)**	**Trig. (mmol/L)**	**HDL (mmol/L)**	**LDL (mmol/L)**	**Ca (mmol/L)**	**P (mmol/L)**	**Na (mmol/L)**	**K (mmol/L)**	**Cl (mmol/L)**
**Control**	1.5±0.4	0.5±0.1	1.1±0.2	0.1±0.1	2.6±0.1	2.8±0.1	144.2±1.8	5.8±0.4	100.9±1.2
**(FD2)H**	1.5±0.2	0.4±0.1	1.1±0.2	0.2±0.1	2.5±0.1	3.0±0.3	143.5±2.3	6.0±0.7	101.2±1.2
**(FD2)L**	1.6±0.3	0.5±0.1	1.2±0.2	0.2±0.2	2.5±0.1	2.9±0.1	144.4±1.0	6.1±0.2	101.3±1.6
**(HM3)H**	1.9±0.3	0.5±0.0	1.5±0.3	0.2±0.1	2.6±0.1	3.1±0.8	144.2±1.9	6.5±1.0	101.3±2.5
**(HM3)L**	1.4±0.2	0.5±0.1	1.1±0.2	0.1±0.1	2.5±0.1	2.7±0.2	143.1±1.5	6.0±0.3	102.0±2.2
**(HM3+FD2)H**	1.6±0.3	0.6±0.1	1.2±0.3	0.2±0.1	2.5±0.1	2.7±0.1	143.1±1.4	6.0±0.5	98.8±1.0
**(HM3+FD2)L**	1.4±0.3	0.5±0.1	1.0±0.3	0.2±0.1	2.5±0.1	3.0±0.2	143.2±2.2	6.1±0.5	101.5±2.2

^1^Values are mean ± SD of 6 replicates

Control, normal saline; (FD2)L, normal saline + 1 × 10^9^ CFU of *L*. *buchneri* FD2/kg BW; (FD2)H, normal saline + 1 × 10^10^ CFU of *L*. *buchneri* FD2/kg BW; (HM3)L, normal saline + 1 × 10^9^ CFU of *L*. *fermentum* HM3/kg BW; (HM3)H, normal saline + 1 × 10^10^ CFU of *L*. *fermentum* HM3/kg BW; (FD2+HM3)L, normal saline + 1 × 10^9^ CFU of a mixture (1:1, w:w) of *L*. *buchneri* FD2 and *L*. *fermentum* HM3; (FD2+HM3)H, normal saline + 1 × 10^10^ CFU of a mixture (1:1, w:w) of *L*. *buchneri* FD2 and *L*. *fermentum* HM3; ALB, albumin; ALT, alanine aminotransferase; AST, aspartate aminotransferase; ALP, alkaline phosphatase; Creat., creatinine; Glu., glucose; T. bil., total bilirubin; Urea, urea; T. prot., total protein; T. chol., total cholesterol; Trig., triglycerides; HDL, high density lipoprotein cholesterol; LDL, low density lipoprotein cholesterol; Ca, calcium; P, inorganic phosphorus; Na, sodium; K, potassium; Cl, chloride

**Table 9:** Serum biochemical parameters of female rats^1^.

**Treatment**	**ALB (g/L)**	**ALT (U/L)**	**AST (U/L)**	**ALP (U/L)**	**Creat. (μmol/L)**	**Glu. (mmol/L)**	**T. bil. (μmol/L)**	**Urea (mmol/L)**	**T. prot. (g/L)**
**Control**	45.4±3.4	54.4±7.2	100.8±12.2	98.2±6.6	75.7±4.1	7.0±0.4	1.1±0.3	6.2±0.8	68.4±3.6
**(FD2)H**	44.4±3.4	51.5±5.8	107.0±15.7.0	91.5±14.2	73.0±1.7	8.1±1.6	1.3±0.8	6.8±1.2	67.9±3.9
**(FD2)L**	42.2±4.0	56.6±12.3	103.7±14.1	91.2±15.1	74.2±4.8	7.7±1.1	1.1±0.3	6.9±1.7	67.1±5.3
**(HM3)H**	41.3±2.8	57.9±10.8	95.3±15.6	99.4±5.1	75.8±3.1	8.6±1.7	0.9±0.7	6.4±0.7	67.0±6.3
**(HM3)L**	41.8±4.6	57.8±10.6	89.2±11.8	87.0±17.1	75.0±3.2	9.2±3.8	1.6±0.1	6.7±1.1	68.0±4.5
**(HM3+FD2)H**	45.9±4.2	62.4±11.08	89.2±12.6	104.3±7.1	71.0±2.9	9.4±2.0	0.8±0.3	6.2±0.8	67.7±4.1
**(HM3+FD2)L**	45.3±3.4	46.3±1.4	108.8±16.5	98.8±13.6	71.2±6.1	8.0±0.6	1.2±0.4	6.5±0.8	65.1±4.6
**Treatment**	T. chol. (mmol/L)	Trig. (mmol/L)	HDL (mmol/L)	LDL (mmol/L)	Ca (mmol/L)	P (mmol/L)	Na (mmol/L)	K (mmol/L)	Cl (mmol/L)
**Control**	2.3±0.4	0.5±0.1	1.9±0.3	0.3±0.09	2.6±0.1	2.6±0.1	143.0±0.6	5.8±0.3	100.4±1.1
**(FD2)H**	2.0±0.5	0.5±0.1	1.5±0.3	0.3±0.23	2.6±0.0	2.5±0.1	143.7±2.1	5.7±0.3	100.8±1.7
**(FD2)L**	2.1±0.4	0.4±0.1	1.5±0.2	0.3±0.08	2.6±0.1	2.5±0.2	143.7±2.2	6.1±0.5	102.2±1.9
**(HM3)H**	2.2±0.2	0.5±0.1	1.6±0.2	0.4±0.06	2.6±0.1	2.6±0.1	143.8±1.0	5.8±0.3	99.8±2.5
**(HM3)L**	2.2±0.2	0.5±0.1	1.7±0.2	0.3±0.11	2.6±0.1	2.6±0.3	143.8±1.7	5.7±0.6	100.5±1.8
**(HM3+FD2)H**	2.2±0.6	0.5±0.1	1.7±0.4	0.3±0.08	2.5±0.1	2.4±0.2	142.5±1.7	6.0±0.5	100.6±2.9
**(HM3+FD2)L**	2.1±0.5	0.5±0.1	1.4±0.3	0.3±0.04	2.5±0.1	2.4±0.1	142.2±1.0	6.2±0.2	100.9±3.1

^1^Values are mean ± SD of 6 replicates

Control, normal saline; (FD2)L, normal saline + 1 × 10^9^ CFU of *L*. *buchneri* FD2/kg BW; (FD2)H, normal saline + 1 × 10^10^ CFU of *L*. *buchneri* FD2/kg BW; (HM3)L, normal saline + 1 × 10^9^ CFU of *L*. *fermentum* HM3/kg BW; (HM3)H, normal saline + 1 × 10^10^ CFU of *L*. *fermentum* HM3/kg BW; (FD2+HM3)L, normal saline + 1 × 10^9^ CFU of a mixture (1:1, w:w) of *L*. *buchneri* FD2 and *L*. *fermentum* HM3; (FD2+HM3)H, normal saline + 1 × 10^10^ CFU of a mixture (1:1, w:w) of *L*. *buchneri* FD2 and *L*. *fermentum* HM3; ALB, albumin; ALT, alanine aminotransferase; AST, aspartate aminotransferase; ALP, alkaline phosphatase; Creat., creatinine; Glu., glucose; T. bil., total bilirubin; Urea, urea; T. prot., total protein; T. chol., total cholesterol; Trig., triglycerides; HDL, high density lipoprotein cholesterol; LDL, low density lipoprotein cholesterol; Ca, calcium; P, inorganic phosphorus; Na, sodium; K, potassium; Cl, chloride

#### Microbiological analysis of cecal contents

**Fig 8:**
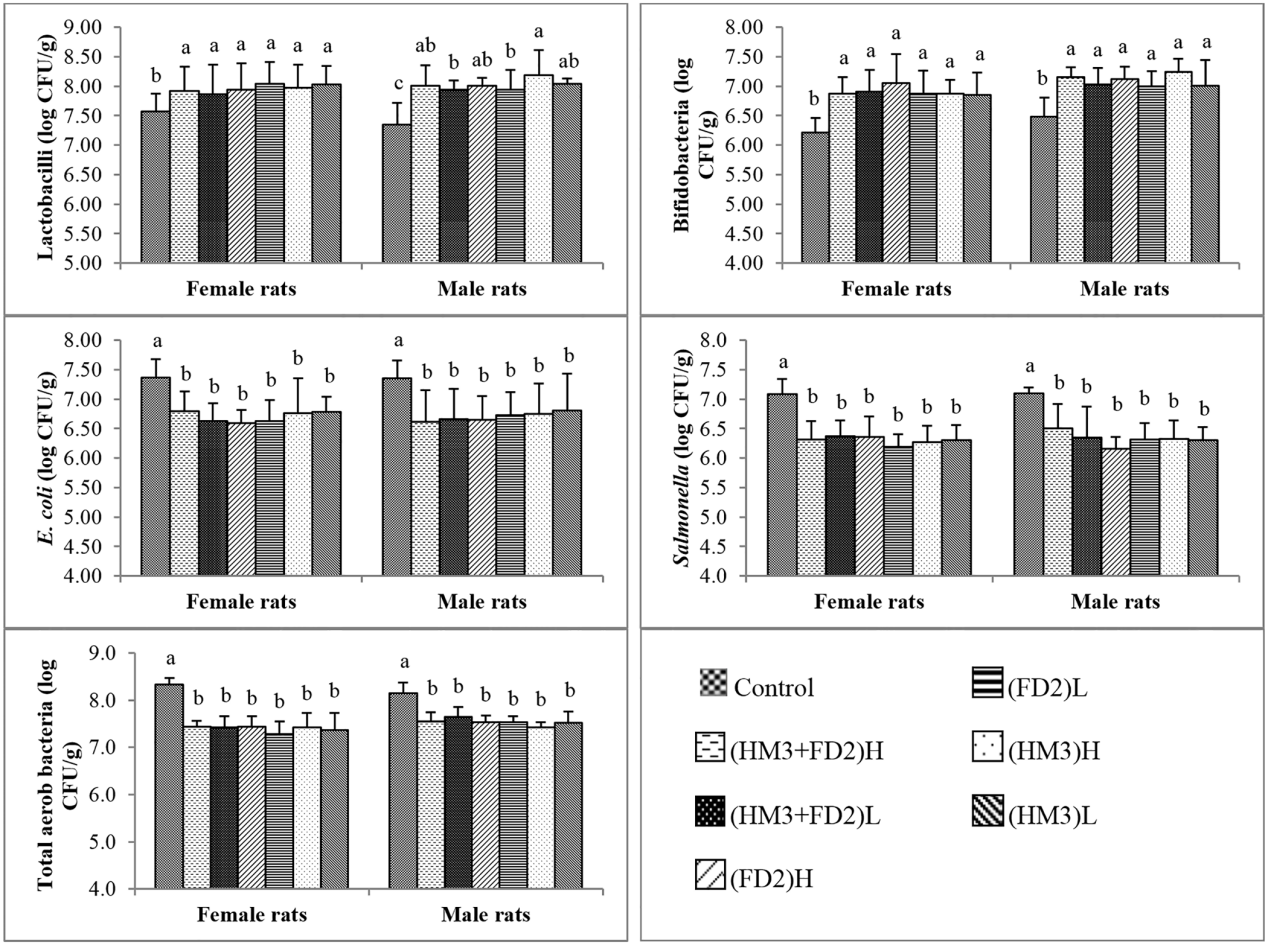
Populations of cecal lactobacilli, bifidobacteria, E. coli, Salmonella and total aerobes of male and female rats. Bars represent means of six rats in each treatment group. Within each gender, bars with different letters differ significantly (P < 0.05). Error bars are standard deviations. Control, normal saline; (FD2)L, normal saline + 1 × 109 CFU of L. buchneri FD2/kg BW; (FD2)H, normal saline + 1 × 1010 CFU of L. buchneri FD2/kg BW; (HM3)L, normal saline + 1 × 109 CFU of L. fermentum HM3/kg BW; (HM3)H, normal saline + 1 × 1010 CFU of L. fermentum HM3/kg BW; (FD2+HM3)L, normal saline + 1 × 109 CFU of a mixture (1:1, w:w) of L. buchneri FD2 and L. fermentum HM3; (FD2+HM3)H, normal saline + 1 × 1010 CFU of a mixture (1:1, w:w) of L. buchneri FD2 and L. fermentum HM3.

The results of cecal bacterial populations of the rates are shown in Fig 8. Results revealed that the cecal populations of lactobacilli and bifidobacteria were significantly (P < 0.05) higher in the treated groups than in the control groups of both male and female rats. For populations of bifidobacteria, there were no significant differences among the treated groups in both male and female rats. However, for populations of lactobacilli, there were no significant differences among the treated groups in female rats, but in male rats, those receiving high dose of HM3 had significantly (P < 0.05) higher population of cecal lactobacilli than those given low dose of FD2 or low dose of HM3+FD2. Cecal populations of *E*. *coli*, *Salmonella* and aerobes were significantly (P < 0.05) reduced in the treated groups when compared to the control groups in male and female rats, and there were no significant differences among the treated groups in both male and female rats.

#### Harmful cecal bacterial enzymes

The results of the harmful cecal bacterial enzymes, β-glucosidase and β-glucuronidase, are shown in Table 10 for both male and female rats. All rats (male and female) in the treated groups had significantly lower (P < 0.01) concentrations of β-glucuronidase in their cecal contents than the control groups. Among the treatments, male rats given high dose of FD2 had significantly (P < 0.05) lower cecal β-glucuronidase concentration than those receiving low dose of HM3 or FD2+HM3, while in female rats, those receiving high dose of FD2 showed significantly (P < 0.05) lower cecal β-glucuronidase concentration than those given low dose of HM3. Although the amounts of β-glucosidase in the cecal contents of all treated groups were lower than the control, they were not significant (P > 0.05).

**Table 10:** β-glucuronidase and β-glucosidase activities in the cecal contents of rats.

	Male rats^1^		Female rats^1^	
Treatment	β-glucosidase (unit/g)	β-glucuronidase (unit/g)	β-glucosidase (unit/g)	β-glucuronidase (unit/g)
**Control**	9.67±0.71	3.87±0.60 ^a^	9.97±0.24	3.79±0.60 ^a^
**(FD2)H**	8.96±0.79	2.22±0.40 ^c^	8.79±1.30	2.46±0.46 ^c^
**(FD2)L**	9.11±0.75	2.51±0.16 ^b^^c^	9.51±1.05	2.73±0.46 ^b^^c^
**(HM3)H**	8.82±1.02	2.50±0.53 ^b^^c^	9.14±0.10	2.61±0.39 ^b^^c^
**(HM3)L**	8.99±1.04	2.83±0.35 ^b^	9.44±1.20	3.10±0.41 ^b^
**(HM3+FD2)H**	8.92±0.77	2.39±0.44 ^b^^c^	8.78±1.30	2.49±0.34 ^b^^c^
**(HM3+FD2)L**	9.16±0.75	2.93±0.56 ^b^	9.43±0.54	3.10±0.41 ^b^^c^

^1^ Values are mean ± SD of 6 replicates

^a–c^ Means within a column with no common superscript differ significantly (P < 0.05)

Unit, the activity required to release 1 μM of p-nitrophenol in 1 h

Control, normal saline; (FD2)L, normal saline + 1 × 10^9^ CFU of *L*. *buchneri* FD2/kg BW; (FD2)H, normal saline + 1 × 10^10^ CFU of *L*. *buchneri* FD2/kg BW; (HM3)L, normal saline + 1 × 10^9^ CFU of *L*. *fermentum* HM3/kg BW; (HM3)H, normal saline + 1 × 10^10^ CFU of *L*. *fermentum* HM3/kg BW; (FD2+HM3)L, normal saline + 1 × 10^9^ CFU of a mixture (1:1, w:w) of *L*. *buchneri* FD2 and *L*. *fermentum* HM3; (FD2+HM3)H, normal saline + 1 × 10^10^ CFU of a mixture (1:1, w:w) of *L*. *buchneri* FD2 and *L*. *fermentum* HM3

## Discussion

Despite many *Lactobacillus* strains are recognized as GRAS [3] and most of them are already used as probiotics in food industry, it has been strongly recommended that new stains should be properly tested for safety before their incorporation into food products [6]. Rat is a standardized physiological and toxicological model of human for different researches because in many cases, its physiology is similar to the corresponding human condition. Hence, usage of rat in researches as a model of human offers many advantages over the mouse and other models [13]. The current study was conducted to assess the safety of two strains of *L*. *fermentum* HM3 and *L*. *buchneri* DF2, and a mixture of them in a rat model using acute oral toxicity and subacute oral toxicity tests.

Acute toxicity study may provide preliminary toxicity data to determine appropriate dose levels for future repeated-dose oral toxicity studies. Such study may also determine possible target organs that have potential to be affected in toxicity studies of a longer duration. Subacute 28-day repeated-dose oral toxicity study provides information on the possible health hazards likely to arise from repeated exposure over a relatively limited period of time.

In the present acute oral toxicity study, there was no mortality in the treated rats. There was also no weight losses, behavior or gross pathological changes or evidence of any sign of toxicity in any of the animals. Since acute toxicity data alone are insufficient for safety assessment of a subject, subacute toxicity or repeated-dose study should be carried out after initial information on toxicity has been obtained by acute toxicity testing (OECD/OCDE guideline, 2008). Hence, a 28-day repeated-dose subacute oral toxicity study with two dose levels of 1 × 10^9^ (low dose) and 1 × 10^10^ CFU/kg BW/day (high dose) of each *Lactobacillus* strain and their mixture (1:1, w:w) was carried out to further investigate their safety aspects.

Based on the results of the repeated-dose subacute oral toxicity study, none of the tested *Lactobacillus* strains caused any sign of toxicity when fed to rats at a concentration as high as 1×10^10^ CFU/kg BW/day (suggested human dose of most probiotics is 10^8^ to 10^9^ CFU). Generally, during the whole period of the subacute oral toxicity study, no toxicological significant differences between the treated groups and the control were observed in terms of body weight, feed consumption, general and behavioral conditions, hematological and serum biochemical parameters, relative organ weights and gross pathological and histopathological changes.

Feed consumption and body weight are good indicators of adverse effects of a substrate in animal studies. Animals which survived cannot lose more than 10% of their initial body weight [14]. In the present subacute oral toxicity study, average weekly feed consumption was similar in all groups in both male and female rats. Body weight was also similar in all groups in female rats, but in male rats, all treated groups at day 28 showed significantly higher body weights than the control group. These results indicated that not only there were no adverse effects on feed consumption and normal growth (body weight) of rats given the two *Lactobacillus* strains, but there were also improved body weights in all male rats given the *Lactobacillus* strains.

Changes in organ weights can be another sign for toxic effects of a test substrate in short-term toxicity tests [15]. In the present study, there were no significant differences in relative weights of organs (brain, heart, lung, liver, spleen, kidney, adrenal, thymus, testes, epididymis, uterus and ovary) among the treated groups, and between all the treated groups and the controls. This indicated that the two *Lactobacillus* strains or a mixture of both strains did not cause any adverse effects on any of the organs. This was further supported by histopathological examinations of the organs which did not revealed any abnormalities nor histopathological changes in the organs.

Hematological and serum biochemical data are critically important for determining toxicological effects induced by treatments. Hematological parameters are usually used for determination of infiammation and infections, and serum biochemical parameters are used to detect organ-related problems [16].

The hematopoietic system is one of the most sensitive targets for toxic substances and is a good measure of the pathological and physiological state in animals and humans. In the current study, results on the hematological parameters demonstrated no significant differences between the treated and control groups. This showed that the two *Lactobacillus* strains did not have adverse effects on the circulating blood cells and their production.

The liver is the main organ in the detoxification and metabolism of chemicals, and AST, ALT and ALP are important liver enzymes that can be used as good indicators for liver function because any damage in the hepatic cells will results in an increase in the serum levels of these enzymes [15]. Bilirubin is another indicator for liver function. A high level of bilirubin is a sign of a problematic liver. According to the American Association for Clinical Chemistry (AACC, 2001), low levels of albumin and total protein are good indicators for problematic functions of both liver and kidney. Also, low level of total protein may occur in inflammatory bowel disease. High level of urea could be a sign of a problematic kidney, and changes in creatinine levels could reflect kidney problems or problems in muscle mass. Damage or swelling of kidney as well as muscle injury could result in high levels of creatinine [17]. High levels of calcium could be due to bone problems (e.g. cancer spread to the bones or broken bones), hyperthyroidism, sarcoidosis or tuberculosis. However, low levels of calcium could be results of underactive parathyroid gland (hypoparathyroidism), acute inflammation of the pancreas, chronic kidney disease, alkalosis and bone disease. Low levels of phosphorus could be related to hypercalcaemia, high levels of parathyriod hormone, low levels of potassium, rickets and osteomalacia. However, higher than normal levels of phosphate (hyperphosphataemia) may be due to kidney failure, underactive parathyroid gland, abnormally low levels of calcium or diabetic ketoacidosis. Other electrolytes like sodium, potassium, and chloride could also be indicators for kidney function. Some forms of heart disease, muscle problems, nerve problems, and diabetes may cause abnormal concentrations of one or more electrolytes in blood serum. High levels of glucose indicate diabetes [17].

Since in the present study there were no significant differences between the treated and control groups in terms of hematological and serum biochemical parameters, it was concluded that the use of the two *Lactobacillus* strains and their mixture at both doses did not have any adverse effects on the internal organ functions.

Another safety concern for probiotic bacteria is the production of potentially harmful intestinal bacterial enzymes such as β-glucosidase and β-glucuronidase [18]. Pathogenic bacteria, particularly *E*. *coli*, produce β-glucosidase and β-glucuronidase. Thus, these two harmful bacterial enzymes are commonly found in the intestines of animals. In the present study, rats receiving the *Lactobacillus* strains had significantly lower amounts of β-glucuronidase than control rats. β-Glucosidase was also numerically lower in the treated rats than control rats. The reduction of these two harmful bacterial enzymes in the treated rats was probably due to the *Lactobacillus* strains. This is shown by the microbiological analysis of cecal contents of the rats. Rats (both male and female) given the *Lactobacillus* strains showed significant increase in the populations of beneficial bacteria such as lactobacilli and bifidobacteria, and a reduction of pathogenic bacteria such as *Salmonella* and *E*. *coli* and total aerobic bacteria when compared to control rats.

## Conclusions

The results of the present study demonstrated that *L*. *buchneri* FD2, *L*. *fermentum* HM3, or a mixture of them were safe up to a level of 10^10^ CFU/kg BW/day in a 14-day or 28-day treatment period. Both strains were well tolerated and there were no observed adverse effects on growth, feed consumption, cellular blood components and vital organs of the treated animals. The *Lactobacillus* strains were also able to reduce harmful intestinal bacterial enzymes, and decrease pathogenic bacterial populations while increasing beneficial bacterial populations. These results suggest that the two *Lactobacillus* strains are safe and could be potential probiotic for human. However, longer chronic oral toxicity studies as well as pre-clinical and clinical trials are required to validate the safety of these two *Lactobacillus* strains before they can be used as probiotics for human consumption.
